# The growth hormone–insulin-like growth factor-I axis in the diagnosis and treatment of growth disorders

**DOI:** 10.1530/EC-18-0099

**Published:** 2018-05-03

**Authors:** Werner F Blum, Abdullah Alherbish, Afaf Alsagheir, Ahmed El Awwa, Walid Kaplan, Ekaterina Koledova, Martin O Savage

**Affiliations:** 1 University Children’s HospitalGiessen, Germany; 2 Al Habib Medical GroupRiyadh, Saudi Arabia; 3 King Faisal Specialist Hospital and Research CenterRiyadh, Saudi Arabia; 4 Department of Pediatric Endocrinology & DiabetesHamad Medical Center, Doha, Qatar; 5 Tawam HospitalAl Ain, UAE; 6 Global Medical Affairs EndocrinologyMerck KGaA, Darmstadt, Germany; 7 William Harvey Research InstituteBarts and the London School of Medicine & Dentistry, London, UK

**Keywords:** growth hormone, paediatrics, short stature, insulin-like growth factor-I, insulin-like growth factor, binding protein-3

## Abstract

The growth hormone (GH)–insulin-like growth factor (IGF)-I axis is a key endocrine mechanism regulating linear growth in children. While paediatricians have a good knowledge of GH secretion and assessment, understanding and use of measurements of the components of the IGF system are less current in clinical practice. The physiological function of this axis is to increase the anabolic cellular processes of protein synthesis and mitosis, and reduction of apoptosis, with each being regulated in the appropriate target tissue. Measurement of serum IGF-I and IGF-binding protein (IGFBP)-3 concentrations can complement assessment of GH status in the investigation of short stature and contribute to prediction of growth response during GH therapy. IGF-I monitoring during GH therapy also informs the clinician about adherence and provides a safety reference to avoid over-dosing during long-term management.

## Introduction

The growth hormone (GH)–insulin-like growth factor (IGF)-I axis is the principle endocrine system regulating linear growth in children (1). Linked to the nutritional status of the individual, GH is a potent stimulator of IGF-I secretion and action. Recombinant human GH (rhGH) administration to children with GH deficiency is effective at promoting growth, and may also enhance height gain in a number of non-GH deficiency disorders (2). Although knowledge of GH secretion and the clinical techniques for assessing GH production are generally well known by practising paediatric endocrinologists, awareness of the physiology of the IGF system and its use in clinical management is less current.

RhGH has been available for clinical use since 1985, when the global Creutzfeldt–Jakob epidemic, related to the use of pituitary-derived hGH, precipitated its licensing by the US Food and Drug Administration and European Medicines Agency. However, rhGH therapy has evolved in the past 30 years. Originally administered intramuscularly three times weekly, it is now given daily by subcutaneous injection.

A number of long-acting GH preparations are currently in various phases of clinical studies (3, 4). Several different techniques have been used in their development, such as modification of the GH molecule by protein enlargement or albumin binding, which results in GH analogues with longer half-lives. Transient binding of GH to polyethylene glycol, so-called pegylation, and use of depot technologies also result in prolonged biological activity. It is likely that weekly or fortnightly GH injections will have an impact on clinical practice in the next 5 years (3, 4).

Understanding of the range of GH responsiveness in GH deficiency, and particularly in non-GH deficiency disorders such as Turner syndrome, short stature related to birth size small for gestational age (SGA) and idiopathic short stature (5), has led to development of prediction models of growth response (6) and a recognition that GH therapy needs to be tailored to each child, depending on their diagnosis and individual characteristics.

Consideration of the IGF system, its contribution to the initiation of GH therapy and the assessment of its effectiveness, has been somewhat neglected. With the aims of studying the GH–IGF-I axis and specifically the use of circulating concentrations of serum IGF-I and IGF-binding protein (IGFBP)-3 in clinical management, an Advisory Board was convened in Dubai in December 2016, by Merck KGaA, Darmstadt, Germany, to address these issues. This article reports the discussions and conclusions of the Advisory Board meeting.

## Physiology of GH–IGF-I axis

### Regulation of GH secretion and targets for GH action

From the age of 1 year onwards, the GH–IGF-I axis is a key regulator of linear growth. Growth hormone is a 191 amino acid protein, with a molecular weight of 22 kDa, produced and secreted in a pulsatile fashion from the anterior pituitary gland (7) under positive control of GH releasing hormone (GHRH) and negative control of somatostatin. Both GHRH and somatostatin are secreted by the hypothalamus. The main targets for GH action are the liver, where it induces the release of both glucose and IGF-I, and adipose tissue, where it controls the release of fatty acids (8). There is also a direct effect on cartilage cells in the growth plates of the long bones, which also secrete IGF-I to act locally (9).

### Components of the IGF system

**Figure 1:**
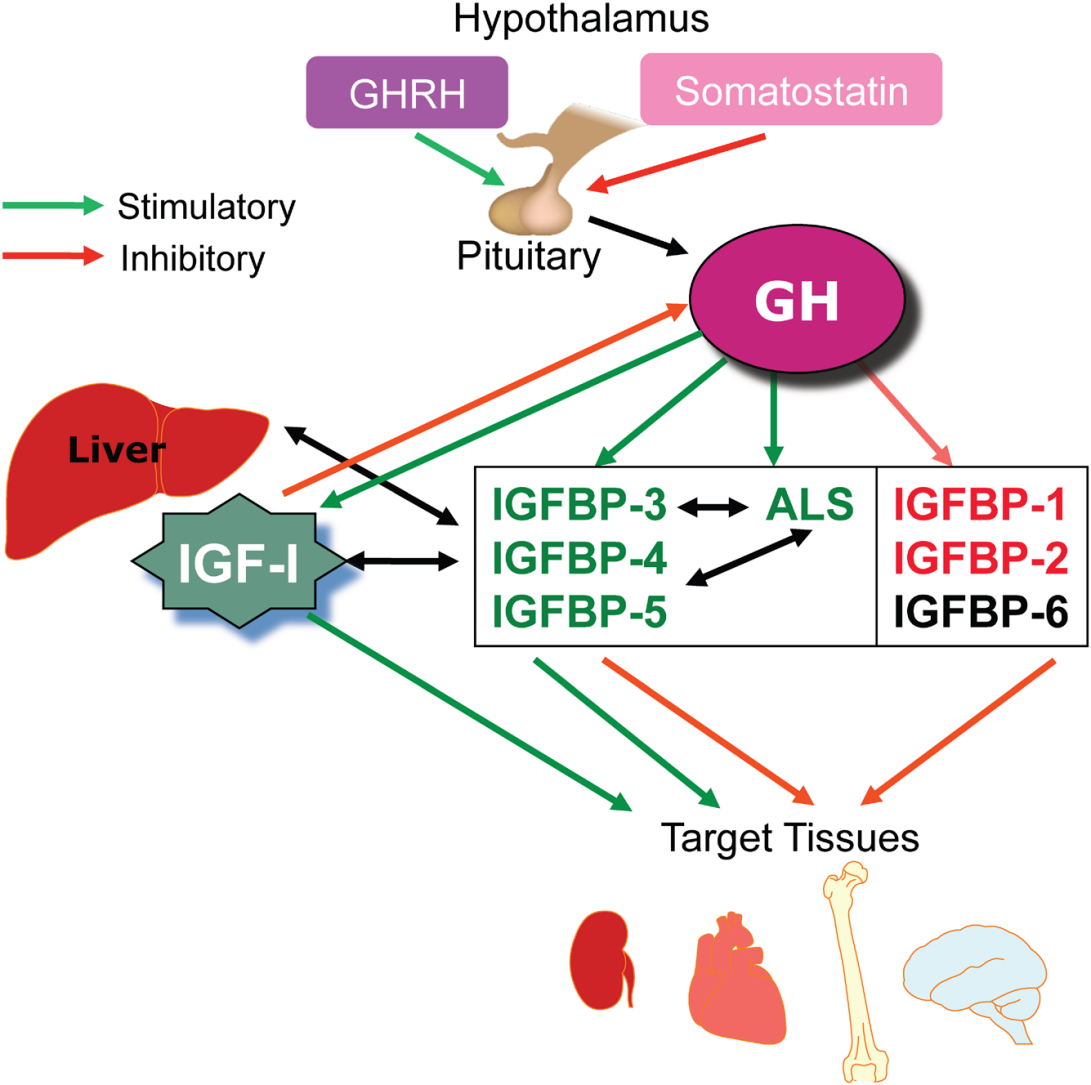
The complex influence of GH through the IGF system. GH, growth hormone; GHRH, GH releasing hormone; IGF, insulin-like growth factor; IGFBP, IGF-binding protein.

**Figure 2:**
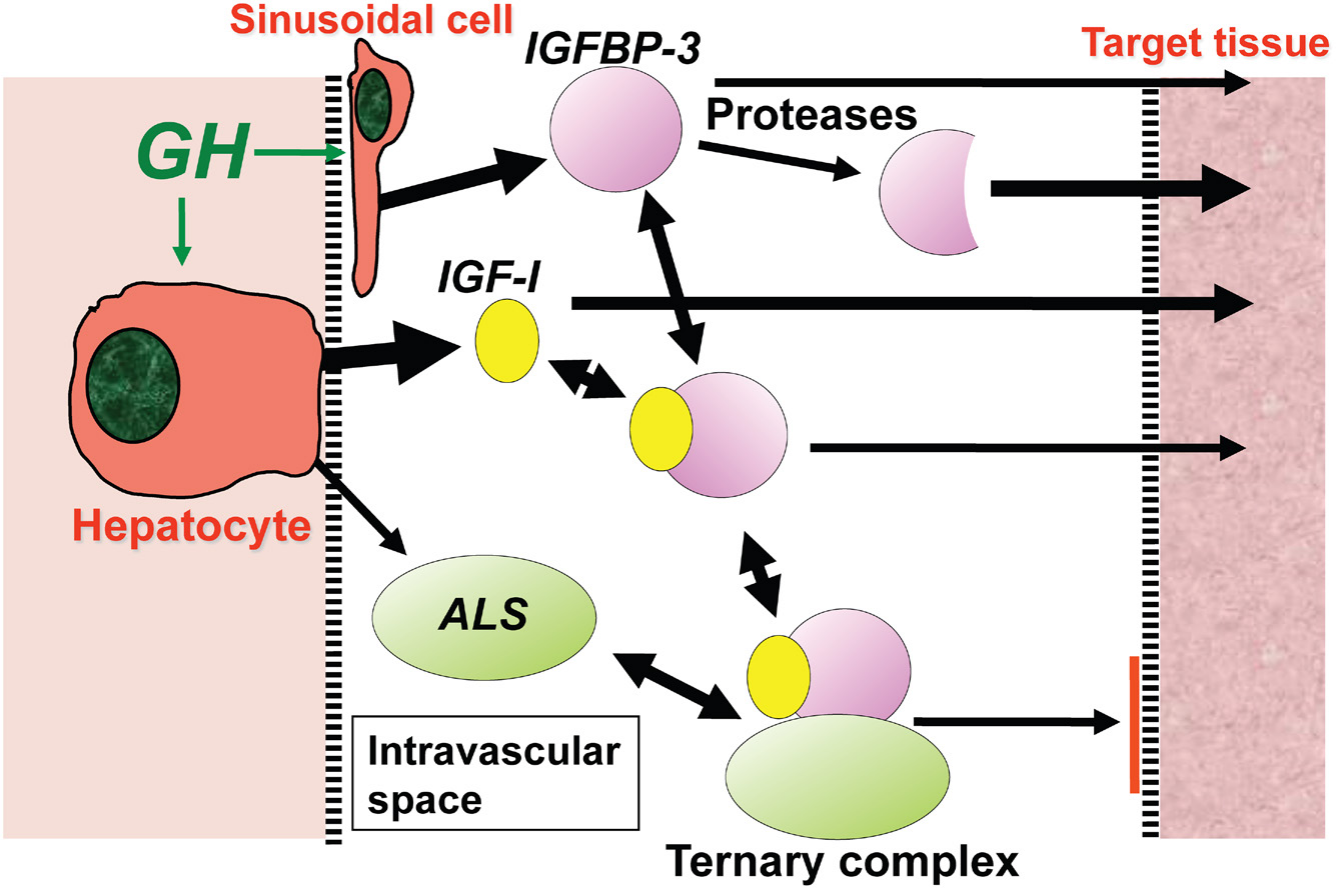
Influence of GH on the generation of IGF-I and IGFBP-3 from the liver into the intravascular space. Interaction of IGF-I with proteases, IGFBP-3 and ALS. ALS, acid labile subunit; GH, growth hormone; IGF, insulin-like growth factor; IGFBP, IGF-binding protein.

**Figure 3:**
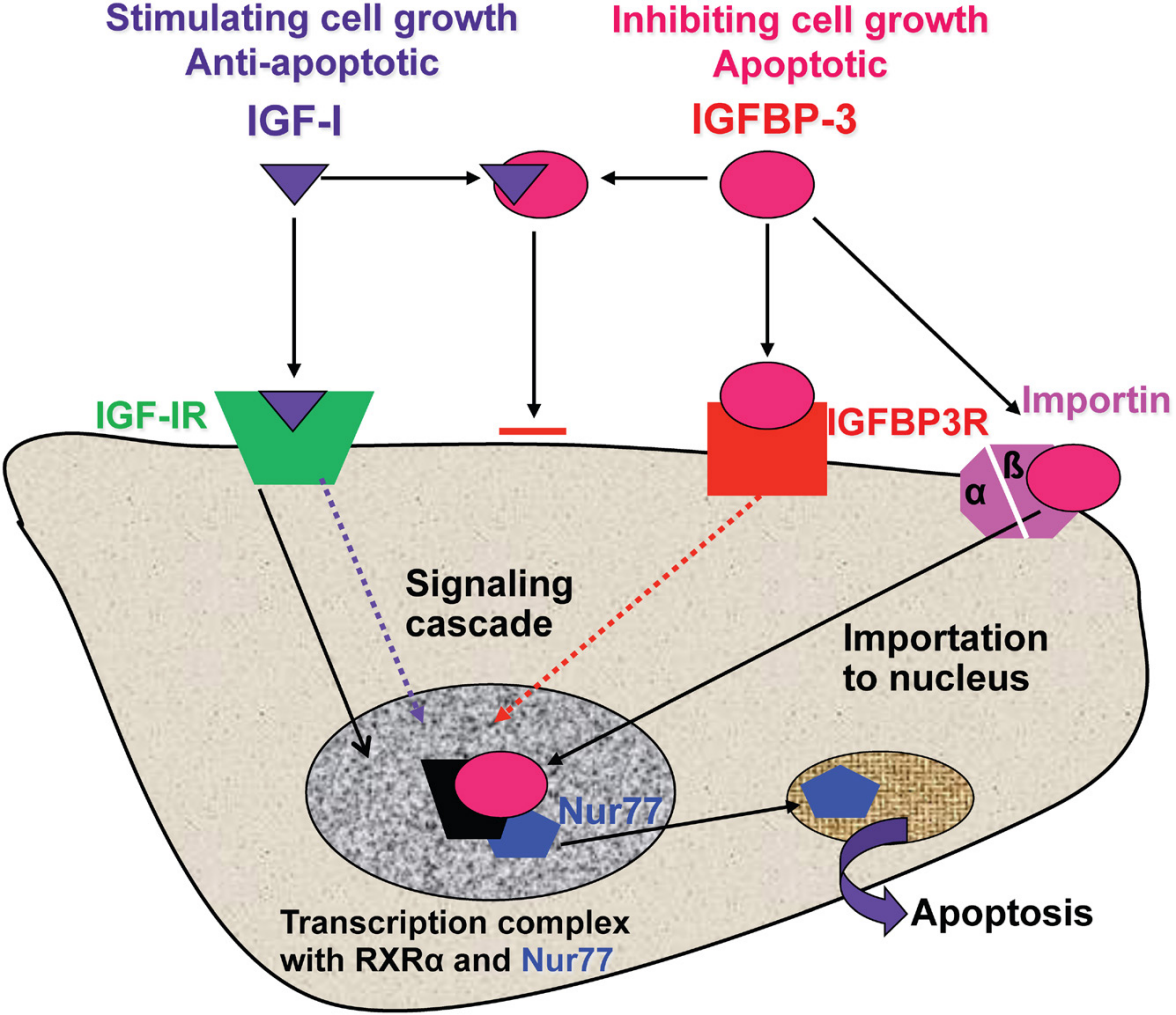
Actions of IGF-I and IGFBP-3 in regulating gene transcription and control of apoptosis. IGF, insulin-like growth factor; IGFBP, IGF-binding protein.

There are numerous components involved in the IGF system. IGF-I itself is a small protein consisting of 70 amino acids, with a molecular weight of 7.65 kDa, and the gene is located at chromosome 12q23 (10). IGF-I in the circulation is predominantly produced by hepatocyte cells in the liver, although it is also secreted by many other cells in the body. IGF-I is bound to specific IGFBPs in the circulatory system, and six IGFBPs have been identified (11). The production of IGFBP-1 and -2 is inhibited by GH whereas production of IGFBP-3, -4 and -5 is stimulated by GH (Fig. 1). IGFBP-3 is produced by the hepatic sinusoidal cells, at the junction of the intravascular space. In the circulation, IGF-I is mainly bound to IGFBP-3 and this binary complex then binds to a large protein called the acid-labile subunit (ALS) to form a ternary complex (1) (Fig. 2). The ternary complex inactivates both IGF-I and IGFBP-3 and prolongs their half-life in the circulation. While IGF-I is bound to IGFBP-3 it is not active, but can be released through chemical equilibrium or proteolysis in peripheral tissues, and free IGF-I then binds to cell surface receptors to trigger a signalling cascade within the cell. However, IGFBP-3 can also act as a hormone, binding either to a specific receptor or to importin-beta at the cell surface. It is then translocated to the cell nucleus where it interacts with the retinoid X receptor and nuclear receptor 77 to form a complex that can regulate transcription and can induce apoptosis (12, 13) (Fig. 3).

### Factors influencing serum IGF-I and IGFBP-3 concentrations

GH is the major stimulator of IGF-I and IGFBP-3 secretion from the liver and levels are decreased by liver failure, returning to normal following restoration of hepatic function (14). However, GH is not the only factor controlling IGF-I concentration in the circulation (Table 1). Insulin (15), thyroid hormone (16) and androgens (17) all stimulate IGF-I release, and oestrogens at low levels stimulate and at high doses inhibit secretion (17). Malnutrition also has a strong inhibitory effect on IGF-I, IGFBP-3 and ALS (18), and serum concentrations are reduced by conditions that affect nutrition, such as coeliac disease (19) and anorexia (20). Malnutrition may act at least partially through an increase in fibroblast growth factor 21, which suppresses GH action and also increases serum IGFBP-1 concentration (18, 21).

**Table 1:** Hormonal regulators of circulating IGF-I and IGFBP-3 concentrations.

Hormone	Effect
Growth hormone	↑↑↑
Prolactin	↑?
Insulin	↑
Thyroid hormones	↑
Glucocorticoids	↑
Androgens	↑
Oestrogens	Low dose ↑, high dose ↓

Gonadotropins, thyroid stimulating hormone, adrenocorticotrophic hormone and parathyroid hormone stimulate local production of IGF-I in their respective target tissues.

IGF, insulin-like growth factor; IGFBP, IGF-binding protein.

Chronic inflammation, as seen for example in Crohn’s disease or juvenile chronic arthritis can disturb the physiological synergy of GH and IGF-I, known as the dual-effector theory. This is based on the principle that GH regulates the expression of locally produced IGF-I, which then acts in an autocrine/paracrine manner. Pro-inflammatory cytokines, such as TNF-alpha, interfere both with circulating IGF-I production resulting in hepatic GH resistance (22) and with the action of locally produced IGF-I by decreasing proliferation and differentiation of chondrocytes in the growth plate (23).

### GH and IGF-I actions on linear growth

The original somatomedin hypothesis proposed that GH did not have any direct effects, but exerted its action to induce bone growth entirely through IGF-I, then known as somatomedin-C (24). However, the ‘dual effector hypothesis’ in 1985 suggested that GH could also act directly to promote differentiation of cells (25). Studies also showed that IGFs are expressed in most tissues and GH regulates locally produced IGF-I, which then acts in an autocrine/paracrine manner; thus, GH and IGFs have complex interconnected direct and indirect actions on the growth plate (10). In the body as a whole, GH and IGF-I appear to have opposite effects on carbohydrate metabolism, whereas lipid metabolism is only affected by GH. Thus, GH causes release of nutrients from stores and IGF-I responds and acts in the tissues where nutrients are needed.

### Growth plate physiology

**Figure 4:**
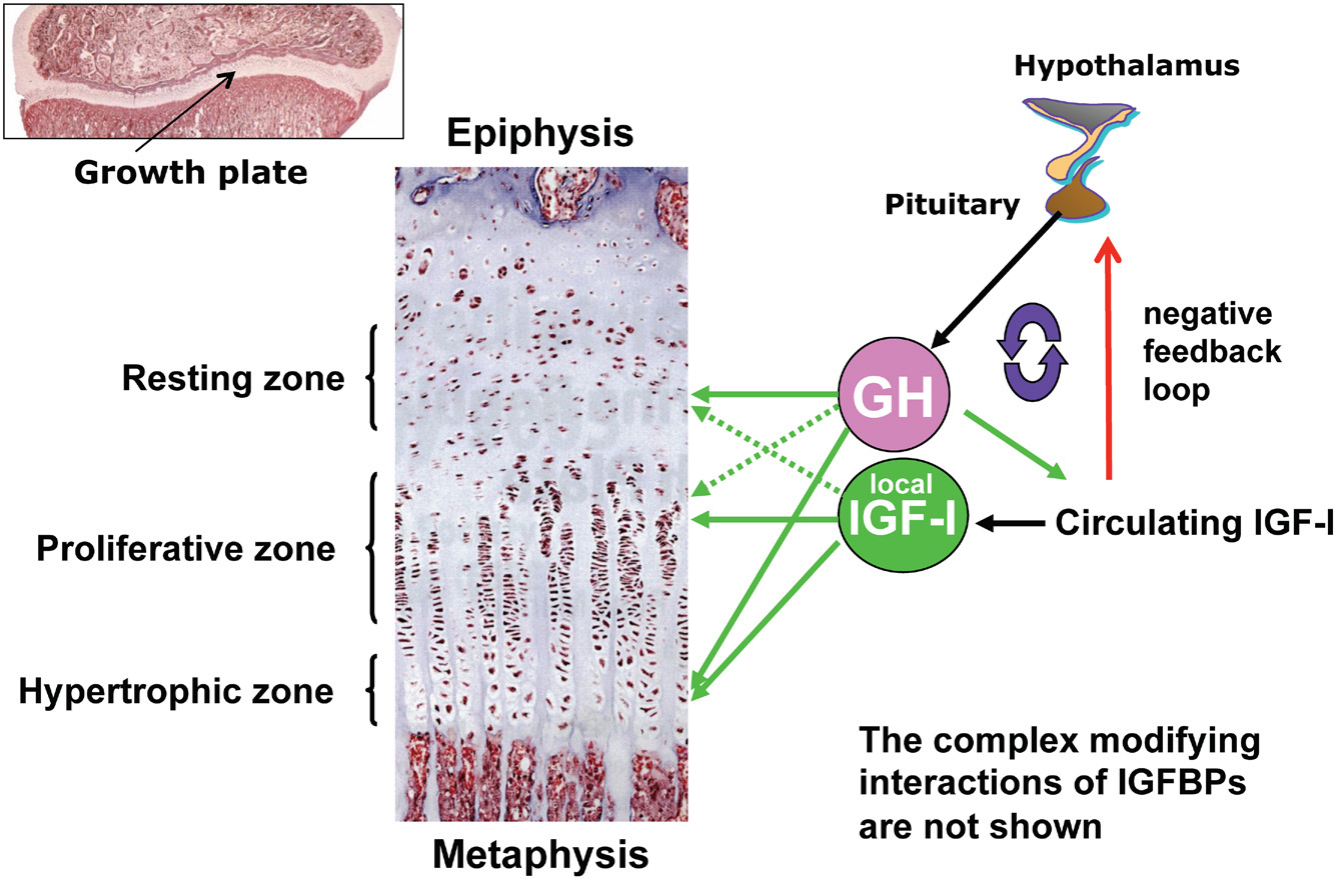
Chondrocyte differentiation, proliferation, hypertrophy and ossification at the growth plate. Reproduced, with permission, from Nilsonn O, Marino R, De Luca F, Phillip M, Baron J. Endocrine regulation of the growth plate. *Hormone Research* 2005 **64** 157–165 (26). Copyright 2005 Karger Publishers, Basel, Switzerland. GH, growth hormone; IGF, insulin-like growth factor; IGFBP, IGF-binding protein.

Bone growth is regulated by a complex network of signals (9). Both GH and IGF-I form part of this network and are able to promote chondrocyte proliferation and differentiation. The cartilage growth plates consist of three main layers known as the resting zone, proliferative zone and the hypertrophic zone, although chondrocytes are the only cell type and each layer is segregated by the rates of proliferation and differentiation within them (9, 26) (Fig. 4). In the resting zone GH is thought to act to induce the chondrocytes, which act like stem cells, to differentiate and proliferate (27). IGF-I acts predominantly at the proliferative and hypertrophic zones to further induce differentiation and increase in height within columns of cells that then calcify the surrounding matrix (26, 28).

### Regulation of pre- and postnatal growth

GH appears to primarily affect postnatal growth and GH deficiency or GH resistance does not significantly affect birth length, weight or BMI (29). In contrast, IGF-I affects both pre-natal and postnatal growth, and defects of IGF-I secretion and action are associated with intrauterine growth retardation and birth size SGA (30, 31). Studies in mice showed that specifically knocking-out liver production of IGF-I resulted in intrauterine growth retardation, but postnatal growth was normal. This was possibly due to GH inducing local production of IGF-I and binding proteins then maintaining a serum pool of IGF-I (8).

### Contributions of circulating vs peripherally secreted IGF-I

Additional knockout of the* IGFALS* gene inhibited linear growth indicating that serum IGF-I, not just locally produced IGF-I, is important to maintain bone growth (32) and deficiency of ALS in children is associated with growth retardation (33, 34, 35). Mice deficient in liver-specific IGF-I also had a large increase in serum GH, which could have contributed to maintain postnatal growth (36). Thus, GH and IGF-I appear to act synergistically, with both involved in bone growth and remodelling, to influence longitudinal growth and bone strength.

## Key steps in the investigation of short stature

### Clinical assessment

For paediatric patients with growth retardation, investigation should begin with clinical assessment that includes family history, medical history, phenotype examination, pubertal stage, measurement of body proportions and assessment for any dysmorphic features (37, 38, 39, 40, 41, 42). Mid-parental height and any available height and weight measurements should be plotted on appropriate charts; World Health Organisation (WHO) charts being frequently used (43), but country-specific charts are preferable because WHO charts are designed for multi-ethnic populations (44). 

### Laboratory assessment

#### General investigations

If the medical history and physical examination do not indicate a specific diagnosis, laboratory tests should be carried out (41, 45), which should include assessments for coeliac disease, Crohn’s disease and determination of karyotype for girls with unexplained short stature and short boys with associated genital abnormalities. While coeliac disease may be considered an uncommon cause of growth retardation, rates of up to 15% of children with short stature have been reported (46, 47) and diagnosis is often delayed (48). Inflammation in children with Crohn’s disease is associated with reduced growth, even after adjustment for steroid use (49). Diagnosis of Turner syndrome in girls with short stature may also be delayed without karyotyping and this may adversely influence morbidity and mortality (50), as well as effectiveness of GH treatment (51, 52). Testing for *SHOX* gene abnormalities should also be considered when the phenotype or family history is suggestive of this genetic defect (37, 53).

#### Endocrine investigations

If no specific diagnosis is identified from general paediatric investigations, the GH–IGF axis should be assessed, starting with measurement of IGF-I and IGFBP-3 concentrations (41). GH stimulation tests should be performed in patients with a medical or familial history and physical examination that is compatible with GH deficiency. However, GH stimulation testing may not be required for patients with hypothalamic–pituitary defects and at least one other pituitary hormone deficiency, where GH deficiency may be assumed (37). A GH stimulation test is also not required for short children with a normal height velocity, no bone age delay and serum IGF-I level at or above the mean for age and gender (5). A peak stimulated GH concentration of <7 μg/L is generally considered to be consistent with GH deficiency (37), however definitions vary from country to country and the biochemical cut-off, e.g. <7 or <10 μg/L, should always be supported by auxological evidence of subnornal growth velocity.

The GH stimulation tests currently recommended for paediatric patients include glucagon, clonidine, arginine or GHRH plus arginine. Interpretation of peak GH concentration should take account of day-to-day variation and impact of nutritional status, age and pubertal development (37, 54). Sex steroid priming is recommended before GH stimulation in children who are just pre-pubertal and with age greater than 10 years in girls and greater than 11 years in boys (37). The peak GH concentration used to define GH deficiency varies by country, by clinical centre and by assay used, and GH assays should use an established international standard (55). Clinicians need to be aware of the assay used in their laboratory and how these factors may affect the response (56, 57, 58).

### Measurement of IGF-I and IGFBP-3 concentrations

IGF-I and IGFBP-3 concentrations can be measured in either serum or plasma samples, but for comparison between patients the same medium should be used to avoid variation; in either case, separation from whole blood should be carried out within 2 h and samples can then be frozen, where determinations remain constant over periods of several years (59). IGF-I and IGFBP-3 concentrations are most frequently measured using immunoassays (60).

Commercially available IGF-I assays, as used in most hospital laboratories, rely on binding of the IGF-I to an antibody, and the antibodies used differ in their ability to bind to IGF-I resulting in substantive variations between assays (55). The antibody should be very specific, with high affinity for IGF-I and minimal cross-reactivity with IGF-II. IGFBPs interfere in the binding of IGF-I antibody (61, 62, 63). To overcome this, serum samples are acidified and an excess of IGF-II is added, with neutralisation of the solution. The IGFBP binding site is blocked by IGF-II, leaving the IGF-I free to bind to the antibody. A number of immunoassays for measurement of IGFBP-3 are commercially available (60, 61, 64, 65); these are unaffected by presence of IGF-I and do not require an acidification step.

**Figure 5:**
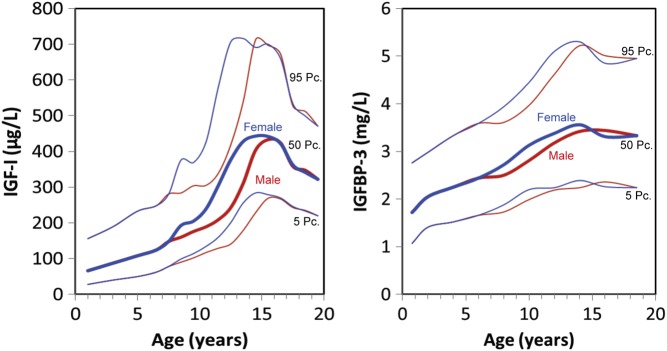
Normal ranges of serum IGF-I and IGFBP-3 in children and interpretation of serum IGF-I and IGFBP-3 levels. Drawn from data reported in Blum WF, Böttcher C, Wudy SA. Insulin-like growth factors and their binding proteins. In: *Diagnostics of Endocrine Function in Children and Adolescents*, ed. 4, pp 157–182. Eds Ranke MB, Mullis P-E. S Karger, Basel, 2011 (61). IGF, insulin-like growth factor; IGFBP, IGF-binding protein.

Serum concentrations of IGF-I and IGFBP-3 show little circadian variation, so measurements can be made from a single sample taken at any time throughout the day. Assays may vary widely in the results for absolute concentration and, therefore, comparing values measured with different assays requires caution. Attempts have been made to cross-calibrate different assay kits for IGF-I and IGFBP-3, which allows conversion of results to make them comparable (66, 67, 68). However, even using the same assay, age, sex and pubertal stage all influence IGF-I and IGFBP-3 concentrations (Fig. 5). Therefore, values from a large reference population are necessary to provide meaningful evaluation of an individual patient against normative data (59, 61, 65, 68, 69). These reference population values can then be used to convert absolute concentrations of IGF-I and IGFBP-3 into SDS (61, 65). Methods for calculating age and sex-based SDS have been described (61, 68), and computer software programs have been developed to enable these calculations to be carried out quickly and easily. Normal values for IGF-I SDS and IGFBP-3 SDS range from −2 to +2, with the 50th percentile as 0.

### Interpretation of IGF-I and IGFBP-3 concentrations

Decreased serum IGF-I SDS is associated with a variety of clinical conditions, in addition to GH deficiency (Table 2). A decrease must, therefore, be considered in relation to other clinical factors when making a diagnosis for an individual patient. Both IGF-I and IGFBP-3 can help distinguish short stature due to GH deficiency from other conditions. In patients younger than 8 years of age, IGF-I levels are relatively low, making it difficult to discriminate between normal and subnormal values. In these young patients IGFBP-3 may provide a better indicator of GH deficiency than IGF-I SDS (61).

**Table 2:** Clinical conditions with decreased IGF-I concentration.

Significantly diminished	Moderately diminished
GH deficiency, including neurosecretory dysfunction and psychosocial growth retardationGH receptor defect (Laron syndrome) and post-GH receptor defectsBioinactive GH (very rare)Neutralising antibodies to GHMalnutrition, malabsorptionLiver insufficiencySevere illness (e.g. sepsis, cachexia, systemic inflammatory disease and malignant disease)Severe trauma, including surgical traumaIGF-II-producing tumours (very rare)	GH deficiency in the presence of severe obesity (e.g. Prader–Willi syndrome)GH deficiency after treatment for malignancyDiabetes mellitusHypothyroidismGH insufficiencyConstitutional delay of growth and adolescence (normal IGFBP-3)Sex steroid deficiency (e.g. Turner syndrome at the age of puberty)

GH, growth hormone; IGF, insulin-like growth factor; IGFBP, IGF-binding protein.

#### The IGF-I generation test

The IGFGT was designed in the 1980s to predict growth responses to GH in patients with GH deficiency. At the time however, this was considered unsatisfactory, the test was abandoned and normative data were not established. Interest in the IGFGT was renewed when patients with short stature associated with GH resistance were selected for recombinant human IGF-I therapy. As the spectrum of short stature disorders grew over time, with many associated with some degree of GH resistance, the criteria of absent responses of IGF-I and IGFBP-3 became too strict for paediatric patients with mild GH resistance or idiopathic short stature (70). Attempts to refine the IGFGT for investigation of growth failure demonstrated that subnormal IGF-I responses were seen in patients with idiopathic short stature; however, additional sensitivity for the diagnosis of GH resistance was not seen with a low-dose GH protocol (71). For these reasons, the IGFGT is not recommended in the routine investigation of short stature (70), although it may support the decision regarding IGF-I treatment in patients with severe GH resistance.

#### IGF-I and IGFBP-3 in GH deficiency

While IGF-I and IGFBP-3 concentrations are only an aid to identify GH deficiency, they do indicate whether a particular GH deficient patient will benefit from GH therapy. The gain in height velocity during GH treatment is significantly correlated with the pre-treatment IGF-I and IGFBP-3 levels. Patients with the lowest levels will generally have a better response to treatment (72).

GH deficiency can be due to many causes and the response to treatment differs according to the aetiology. For approximately 80% of patients the cause is not specifically identified and is reported as idiopathic GH deficiency (41). Structural defects are most commonly associated with pituitary abnormalities, which can be identified by magnetic resonance imaging (73, 74). Such patients have very low serum IGF-I SDS and the level depends on the severity of the condition. Acquired GH deficiency occurs less frequently, accounting for approximately 20% of cases. It may result from brain tumours following radiotherapy affecting the central nervous system.

#### IGF-I and IGFBP-3 in non-GH deficient short stature

IGF-I SDS and IGFBP-3 SDS below −2 would indicate an abnormality in the GH axis, provided that other factors, such as malnutrition, severe illness and trauma, have been eliminated. In patients with non-GH-deficient short stature, IGF-I SDS and IGFBP-3 SDS values generally do not contribute to the diagnosis. In patients with idiopathic short stature, without evident GH deficiency and no other cause identified, approximately 40% may have IGF-I SDS below the normal range (61). Patients with other causes of short stature, such as Turner syndrome, born SGA without catch-up growth and abnormalities of the short stature homeobox-containing gene (*SHOX*), generally have IGF-I SDS within the normal range. However during GH therapy in Turner syndrome and SGA, IGF-I SDS values of >2 may be seen and require monitoring. Prolonged elevation of IGF-I may indicate the need to decrease the dose of GH.

The combination of short stature and IGF-I levels close to or above the upper limit of the normal range should trigger particular attention to the phenotype and a possible history of low birth weight. IGF-I receptor defects combine low birth weight and high normal IGF-I values (1). The recently reported mutations of the proteolytic factor PAPP-A2 are associated with a short stature phenotype which may be subtle, but includes facial and skeletal dysmorphism and IGF-I, IGFBP-3 and ALS values that are pathologically elevated (75).

## IGF-I and IGFBP-3 measurements during GH therapy

### Use of IGF-I and IGFBP-3 for initiation and evaluation

During GH therapy in patients with short stature, monitoring of IGF-I SDS and IGFBP-3 SDS can be used for testing compliance and guiding GH dose adjustments. Evaluation of safety is also an important role for IGF-I monitoring, with the aim being to avoid increases above the normal range. IGF-I levels at baseline and changes during GH therapy are used in prediction models of the growth response to GH therapy (6, 72). After starting GH therapy, IGF-I takes 3 to 5 days to increase sufficiently for monitoring of responsiveness (76). Restoration of IGF-I SDS within the normal range has been suggested when monitoring effectiveness of GH therapy in children. However, for children with GH deficiency, GH therapy is effective even when IGF-I SDS is not normalised, particularly in patients with severe GH deficiency when pre-treatment IGF-I SDS is very low (77).

### Epidemiological studies of high IGF-I and IGFBP-3

Epidemiological studies in adults have suggested that high IGF-I concentrations could potentially be associated with certain cancers; however, opposite effects have been reported for high IGFBP-3. Hypothetically, assessment of possible risk can be identified using tertiles of IGF-I SDS and IGFBP-3 SDS. Patients with levels in the possible risk area of high IGF-I SDS with concomitant low IGFBP-3 SDS may require a reduction in GH dose in order to avoid any potential risk of adverse events (61).

## Conclusions

In the healthy child, the GH–IGF-I axis drives the mechanisms of anabolism and linear growth. Physiologically, IGF-I provides the message to cells that nutrition and general health are appropriate for enhanced protein synthesis, increased mitosis and avoidance of apoptosis in response to stimuli. Each process is regulated by GH, IGF-I and IGFBP-3, together with insulin, in the appropriate target tissue. Assessment of GH secretory capacity is a key stage in the investigation of short stature and is generally recommended, except in the child with normal serum IGF-I and normal height velocity. The use of measurements of serum IGF-I and IGFBP-3 concentrations is also recommended, but has been hindered by assay difficulties. In GH deficiency, low IGF-I and IGFBP-3 levels complement the finding of diminished peak GH in response to provocative testing. IGF-I and IGFBP-3 measurements can inform the clinician about adherence to GH treatment and, if maintained at <+2 SDS, will generally avoid GH over-dosage.

As explained above, multiple factors can influence serum IGF-I and IGFBP-3 concentrations, hence they are not the pure guide to GH secretion or action. Nevertheless, they are valuable investigative and therapeutic tools which contribute to the variables available to clinicians for the management of growth failure in children.

## Declaration of interest

E K is an employee of Merck KGaA, Germany. The remaining authors declare that there is no conflict of interest that could be perceived as prejudicing the impartiality of this review.

## Funding

This work was supported by Merck Serono Middle East FZ-LLC, Dubai, UAE, an affiliate of Merck KGaA, Darmstadt, Germany.
